# A Case of Intraocular Proliferative Changes Caused by a Glaucoma Tube Device

**DOI:** 10.7759/cureus.26445

**Published:** 2022-06-30

**Authors:** Fumiya Miyako, Yoshiaki Kiuchi, Hiromitsu Onoe, Naoki Okada, Hideaki Okumichi, Kazuyuki Hirooka

**Affiliations:** 1 Ophthalmology, Hiroshima University, Hiroshima, JPN

**Keywords:** glaucoma tube device, glaucoma surgery, foreign body reaction, ahmed, pediatric glaucoma

## Abstract

In recent years, glaucoma tube surgery has been recommended for refractory cases that have failed to respond to angle surgery. In this study, we described the case of the fibrous proliferative membrane caused in the anterior chamber after Ahmed glaucoma valve implantation in a pediatric glaucoma patient. He was born full term, weighing 3228 g. Corneal opacity in both eyes was seen at birth and he was referred to the Department of Ophthalmology, Hiroshima University Hospital on the 13th day of his life. At the initial examination, the intraocular pressure was 37mmHg right 25mmHg left. Corneal diameter expansion and diffuse corneal opacity were seen in both eyes. Nine days after the initial examination, trabeculotomy was performed in both eyes but they were ineffective, and Ahmed glaucoma tubes were inserted in both eyes two months later. Four months later the intraocular pressure remained 30mmHg range in both eyes and micropulse cyclophotocoagulation was performed. One year after the Ahmed glaucoma valve implantation, the tube of right eye was exposed, and we planned to perform a repair procedure. At this time, ultrasound biomicroscopy (UBM) showed proliferative tissue around both tubes. They were removed next month. Although silicone is a highly biocompatible material, it can cause foreign body reactions such as encapsulation around the silicone plate and proliferative membranes around silicone oil. We speculated that a similar reaction occurred to the silicone tube in this case. We reported a case of fibrous proliferative membrane in the anterior chamber. This might be caused by the silicon tube of the Ahmed glaucoma valve.

## Introduction

In recent years, tube surgery has been recommended for refractory cases that have failed to respond to angle surgery. The main complications of tube surgery are tube malposition, tube exposure, endophthalmitis, corneal endothelial damage caused by the tube contacting the corneal endothelium, strabismus, and motility limitations [1].

In this study, we report a case of fibrous proliferative membrane in the anterior chamber after Ahmed glaucoma valve implantation in a pediatric glaucoma patient who did not respond to trabeculotomy.

## Case presentation

A 13-day-old boy was referred to the Department of Ophthalmology, Hiroshima University Hospital. Main complaint: Corneal opacity in both eyes. Present illness: Corneal opacity in both eyes was seen at birth. His parents didn’t refer any other symptoms. Family history: Nothing to report. Visual acuity: Unevaluable. Corneal diameter expansion and diffuse corneal opacity in both eyes were seen. Intraocular pressure (IOP) was 37mmHg right and 25mmHg left with iCare® (Raleigh, NC, USA). Palpation showed both eyes rock hard.

**Figure 1:**
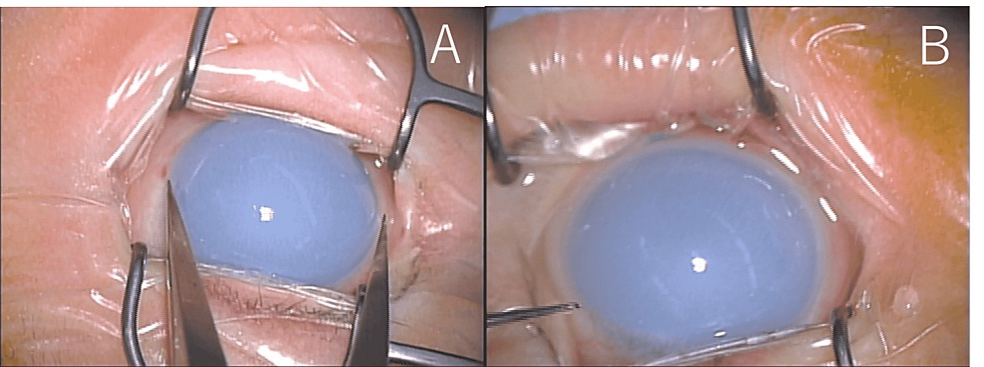
A picture of anterior segment under general anesthesia before trabeculotomy at 22 days of age A; right eye. B; left eye. The corneal diameter was 11.75 mm in the right eye and 12.25 mm in the left eye, and there was diffuse corneal opacity in both eyes. Haab lines could not be observed.

Nine days after the initial examination, the patient underwent a thorough examination under general anesthesia with sevoflurane. IOP was 23.5mmHg right eye, 36.0mmHg left eye with iCare®. The corneal diameter was 11.75 mm right and 12.25 mm left, and diffuse corneal opacity was seen in both eyes (Figure 1).

Haab lines could not be observed. The fundus was not observed due to corneal opacity. The axial length was 21.64 mm right, 21.99 mm left (the average axial length of newborns is 18.6 mm) [2]. Ultrasound biomicroscopy (UBM) showed an increase of corneal thickness in both eyes, with 0.9 mm on the right and 1.1 mm on the left. No other remarkable changes were detected by ultrasonography. We diagnosed congenital glaucoma and performed trabeculotomy ab externo in both eyes on the same day.

Despite the trabeculotomy, IOP remained elevated. Bilateral Ahmed glaucoma valve implantation was performed two months later in both eyes. After insertion, IOP remained 30mmHg range in both eyes so micropulse ciliary photocoagulation (MPCPC) was performed four months later. At the time of the pre-laser examination under general anesthesia with sevoflurane, IOP was 13mmHg in the right eye and 30mmHg in the left eye with iCare®. There was no significant change in corneal opacity or UBM findings. 

Although IOP decreased in both eyes to the 10mmHg range one week after MPCPC, IOP in the left eye elevated to 34mmHg one month later, right eye remained 10mmHg with iCare®. We initiated tafluprost eye drop once a day in the left eye. Three months after MPCPC, IOP in both eyes was 10mmHg, under tafluprost eye drop once a day in the left eye.

**Figure 2:**
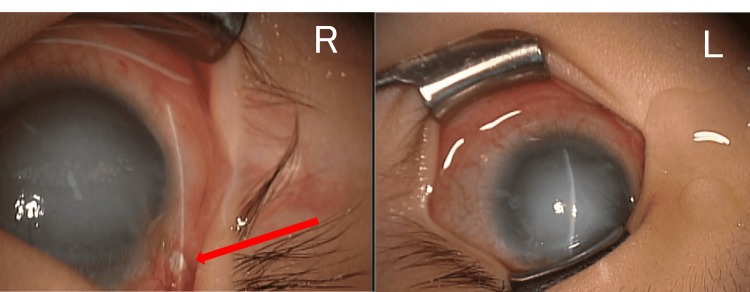
Pictures of anterior segment at the tube covering procedure under general anesthesia. A; right eye, B; left eye. There was a tube exposure in the supra temporal area (red arrow) of the right eye and no tube exposure in the left eye.

On examination seven months after MPCPC, enophthalmos and tube exposure of right eye was seen. The IOP was 19mmHg in the left with iCare® but the IOP of the right eye could not be measured. The palpation showed the tone of right eye was soft. We guessed that enophthalmos was due to a leak from the tube exposure and decided to perform a covering procedure (Figure 2).

**Figure 3:**
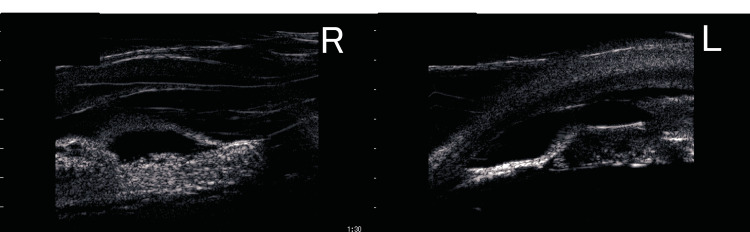
Ultrasound biomicroscopy (UBM) image at the tube covering procedure under general anesthesia. A; right eye B; left eye. The proliferative membrane in the right eye extended from the posterior chamber to the posterior surface of the cornea, while in the left eye, it was localized around the tube.

At the preoperative examination, UBM showed fibrous proliferative membrane in both eyes. The proliferative membrane in the right eye extended from the posterior chamber to the posterior surface of the cornea, while in the left eye, it localized around the tube (Figure 3).

B-mode scan showed diffuse hyperintense echogenicity in the vitreous cavity of the right eye, suggestive of vitreous hemorrhage, and the axial length of the right eye looked shorter than that of the left eye. The IOP of the left eye was 28mmHg with Tonopen® but that of the right eye could not be measured. The palpation showed the right eye soft. Based on the findings of UBM, we guessed that proliferative membrane reached the backside of the iris and ciliary body and ciliary body aqueous humor formation may be decreased due to proliferative membrane. We worried that the same process would occur in the left eye, so we performed Ahmed extraction in both eyes. Brimonidine tartrate and brinzolamide combination twice daily and carteolol hydrochloride and latanoprost combination were administered in the left eye on the next day of the surgery because we assumed that the IOP in the left would be elevated after Ahmed extraction.

One month after the extraction surgery, IOP was 10mmHg in the right eye and 11.5mmHg in the left eye under IOP reduction eye drops in the left eye. Two months after Ahmed extraction surgery, the left IOP elevated to the 50mmHg range while right was 6mmHg and we performed MPCPC in the left eye. One month after MPCPC, the left IOP was 25mmHg under the same eye drops.

Parents are reluctant to add further surgical treatment.

## Discussion

Diseases presenting with congenital corneal opacity include congenital corneal dystrophy, Peter’s anomaly, dermoid, scleral cornea, congenital glaucoma, metabolic diseases such as cystinosis and mucopolysaccharidosis, trauma and infection [3]. In this case, there was no history of trauma or infection, and there were no abnormalities in the iris or lens. Moreover, the corneal opacity did not improve when the IOP was lowered, and there were no systemic abnormalities other than the eyes. The corneal opacity was due to congenital corneal endothelial dystrophy. Congenital glaucoma coexistence with congenital corneal endothelial dystrophy was previously reported [4]. It may be possible, but corneal edema of prolonged duration, as seems to be the case here, since IOP remained elevated despite trabeculotomy and Ahmed valve implantation, can also cause permanent corneal opacity.

Silicone is highly biocompatible and used in ophthalmic surgery for glaucoma valve implants and silicone oil injected into the vitreous cavity for retinal detachment [5,6]. However, foreign body reactions to silicon can also occur. Hilel et al. [7] reported the recurrence of tractional retinal detachment due to the formation of a proliferative membrane around silicone oil injected for retinal detachment. They did not describe the mechanism of formation of proliferative membrane [5]. It has been reported that even the inner ear, which is thought to be as immune tolerant as the anterior chamber, can show unusual reactions. In cochlear implants, granulomatous changes have occurred around silicone tubes inserted into the cochlea [7].　

In general, a foreign body reaction occurs in vivo when an artificial material is implanted in a living body. Plasma proteins are adsorbed on the surface of the material, followed by the fusion of monocytes and macrophages to form foreign body giant cells [6]. In addition, plasma proteins adsorbed on the silicon surface form a 'protein matrix' that triggers the immune system. Then a variety of cytokines, chemokines, and growth factors are released to activate immune cells. This inflammatory response creates a microenvironment that contains tumor necrosis factor (TNF)-α and induces the maturation of extracellular matrix proteins in fibroblasts, leading to fibrosis [8].　 

## Conclusions

The anterior chamber is immune tolerant and silicon is a highly biocompatible material. Foreign body reactions rarely occur in the anterior chamber by silicon tubes. In this case the patient was a child. A special immune response may have occurred in the anterior chamber.

We report that fibrous proliferative membrane can occur in the anterior chamber after Ahmed glaucoma bulb insertion. When performing tube insertion surgery, attention should be paid to proliferative changes as well as IOP.
